# Unravelling genetic etiology of cerebral palsy: findings from a Slovenian pediatric cohort

**DOI:** 10.3389/fneur.2025.1615449

**Published:** 2025-07-23

**Authors:** Ula Arkar Silan, Ana Trebše, Jernej Kovač, Mihael Rogac, Anja Troha Gergeli, Robert Šket, Tina Bregant, David Neubauer, Borut Peterlin, Damjan Osredkar

**Affiliations:** ^1^Department of Child, Adolescent and Developmental Neurology, University Children's Hospital, University Medical Centre Ljubljana, Ljubljana, Slovenia; ^2^Faculty of Medicine, University of Ljubljana, Ljubljana, Slovenia; ^3^Clinical Institute of Special Laboratory Diagnostics, University Children's Hospital, University Medical Centre Ljubljana, Ljubljana, Slovenia; ^4^Clinical Institute of Genomic Medicine, University Medical Centre Ljubljana, Ljubljana, Slovenia; ^5^Centre for Education, Rehabilitation and Training - CIRIUS Kamnik, Kamnik, Slovenia; ^6^Faculty of Medicine, Center for Developmental Neuroscience, University of Ljubljana, Ljubljana, Slovenia

**Keywords:** cerebral palsy, genetic etiology, whole exome sequencing, gene therapy, CTNNB1

## Abstract

**Introduction:**

Cerebral palsy (CP) is a permanent movement or postural disorder due to non-progressive injury to the developing brain, with recent research suggesting a genetic contribution in many patients. This study aimed to investigate the genetic etiology of CP in Slovene children without a previously suspected genetic cause or with prior negative genetic testing.

**Methods:**

All children born after 2003 from the Slovenian National Registry of Cerebral Palsy (SRCP) without an established genetic diagnosis were invited to participate in this cross-sectional study. Whole exome sequencing (WES) was conducted, followed by analysis of 110 CP-associated genes. Thirteen patients underwent additional family segregation by Sanger sequencing. Genetic findings were classified according to the ACMG guidelines.

**Results:**

The study included 136 children, of whom 68 (50%) were male. Spastic CP was identified in 85% of the participants, dyskinetic in 13%, and ataxic in 2%. Gross Motor Function Classification System (GMFCS) levels varied, with the majority (36%) classified as level I. Pathogenic variants, likely pathogenic variants, or ‘*de novo*’ variants of unknown significance (VUS) were identified in nine children (6.6%) in *ATL1*, *CTNNB1*, *DYRK1*, *KMT2A*, *PROC*, *SPAST*, *ZC4H2*, and *ZSWIM6*. Among these nine children, two had normal brain Magnetic Resonance Imaging (MRI) and three had an unsuspicious medical history.

**Conclusion:**

This study identified plausible, possible, or definite genetic etiologies in a cohort of children with CP. Apart from the exclusion of individuals with a previously established genetic diagnosis, no other selection criteria were applied, allowing for an inclusive assessment of genetic contributions within this population. With the advent of personalized medicine and genetic treatment, understanding the genetic underpinnings of CP is crucial for targeted therapy.

## 1 Introduction

Cerebral palsy (CP) is the most prevalent physical disability of early childhood that results in a lifelong impairment (1). According to Surveillance of cerebral palsy in Europe (SCPE) (2), CP is defined as a group of permanent, but not unchanging, disorders of movement and/or posture and of motor function, which are due to a non-progressive interference, lesion, or abnormality of the developing/immature brain. In Europe, CP affects roughly 1,8 children per 1,000 live births (3).

Traditionally, CP was believed to be a consequence of birth and early neonatal complications (4, 5). However, recent studies dispute traditional risk factors as the exclusive cause, with up to one-third of CP patients lacking such factors (6). Recent research highlights the possible role of genetic variants in CP, with at least 4% of patients with CP carrying pathogenic copy number variants (CNV) and 14% having pathogenic single nucleotide variants (SNV) or indels (7, 8). These rates may be higher in patients with additional neurodevelopmental abnormalities (7, 8). Two recent meta-analyses (9, 10), which included methodologically diverse studies with heterogeneous patient populations, reported an overall diagnostic yield of genetic testing in CP patients between 23 and 31%, with higher rates observed in cohorts using exclusion criteria for patient selection or patients without compelling risk factors. Increasing evidence suggests that CP is a genetically heterogeneous disorder (11, 12). Each year, new candidate genes are identified in individuals with CP phenotypes, reflecting a rapidly evolving field and contributing to a deeper understanding of the underlying patophysiology (13).

Recently, recognition of genetic causes has gained significant importance because of the emergence of new therapeutic opportunities for children affected by rare genetic diseases. A notable example is aromatic l-amino acid decarboxylase (AADC) deficiency, in which gene therapy, in the form of intraputaminal infusions of a recombinant adeno-associated virus type 2 vector containing the human *AADC* gene, is already accessible (14, 15).

This study aimed to offer genetic testing to children from the Slovenian National Registry of Cerebral Palsy (SRCP) without a previously established causative genetic diagnosis to uncover the genetic etiology of CP and provide genetic counselling to patients and their families.

## 2 Patients and methods

This was a cross-sectional cohort study. All children with CP born after 1996 who have national medical insurance in Slovenia are included in the Slovenian National Registry of Cerebral Palsy (SRCP), which participates in the SCPE. All patients included in the registry were diagnosed with CP by a developmental pediatrician or child neurologist, according to the currently established SCPE definition (2). Children are registered in the SRCP at the University Children’s Hospital, University Medical Centre Ljubljana by a pediatric neurologist at a minimum age of 4 years. The data collected in the registry consists of demographic data, CP classification data, pregnancy and perinatal complications, neuroimaging reports, epilepsy and other associated disorders, current medical care, and socioeconomic data (16, 17).

All patients with CP registered in the SRCP with available contact information, who were born between 1.1.2003 and 1.1.2020 and were at least 4 years of age at the time of enrollment, and did not have an established genetic diagnosis causative of CP, were invited to participate in the study. At the time of study initiation in 2022, 685 children were registered in the SRCP—of these, 459 met the age criterion. Among them, 42 patients had a previously established genetic diagnosis causative of CP (e.g., a pathogenic variant in CP-associated genes) and were therefore excluded from the study. Additionally, 14 patients were registered anonymously and could not be contacted. As a result, 403 patients were eligible for contact and were invited to participate in the study. Patients were contacted by phone when a valid number was available; otherwise, an invitation letter was sent by post. A total of 139 families responded positively and agreed to participate. After excluding three families who later declined participation, 136 children were included in the final cohort.

At enrollment, we actively screened our patients for the presence of “red flags,” which are clinical and imaging signs, raising the suspicion of a genetic origin of the disease in patients with CP. The red flags were normal or nonspecific Magnetic Resonance Imaging (MRI) findings, severe symptoms in the absence of a history of perinatal injury (uneventful pregnancy and perinatal history), positive family history of CP, neurodevelopmental regression, progressively worsening symptomatology (18, 19), paraplegia, rigidity as opposed to spasticity (18), dysmorphic features, fluctuation in motor symptoms, paroxysmal symptoms in relation to time of day, diet/fasting, or activity, peripheral nervous system abnormalities, and eye movement abnormalities (e.g., oculogyric crises) (19).

### 2.1 Genetic testing

Genomic DNA was processed and sequencing libraries were constructed using a tagmentation-based workflow followed by hybrid capture enrichment with an Illumina-compatible kit (Illumina Inc., San Diego, CA, USA) and the IDT xGen Exome Research Panel probes according to the manufacturer’s protocols (Integrated DNA Technologies). The resulting libraries were sequenced on an Illumina NovaSeq 6,000 platform using 150 base pair paired-end reads (2 × 150 bp). Subsequent to this preparation, the size of the fragmented libraries was accurately gauged using the industry-standard Agilent 2,100 Bioanalyzer (Agilent Technologies, Inc., USA). To ensure optimal sequencing, the libraries underwent rigorous quantification using the NEBNext® Library Quant Kit for Illumina (New England Biolabs, Inc., USA) and were then loaded for sequencing at a concentration of 1.1 nM. Raw sequencing reads were quality-checked and then aligned to the human reference genome (hg19) using the Burrows-Wheeler Aligner (BWA-MEM, version 0.7.17). Duplicate reads were marked and removed. Variant calling was performed using the Genome Analysis Toolkit (GATK, version 4.2.0.0) HaplotypeCaller module following the GATK Best Practices workflow (20).

The genome under investigation belonged to *Homo sapiens*. Called variants were filtered to retain those with a minor allele frequency (MAF) of less than 1% in population databases (e.g., gnomAD). Only exonic, nonsense, and canonical splice site variants were included in the downstream analysis. Variant annotations were performed using Annovar. Variants were further restricted to an authoritative list of genes linked to CP. This list of 110 genes was compiled in 2021 using the Human Phenotype Ontology (HPO) database^1^ by searching under the CP phenotype term (HP: 0100021). The full list is available in Supplementary Table 1. Conclusively, from the refined and curated variant list, a candidate variant was identified as being potentially causal to the clinical phenotype exhibited by the participant.

Families of children who received positive genetic results, as well as those with negative results who opted for further consultation, were referred to a clinical geneticist for genetic counseling.

### 2.2 Ethics approval

This study was approved by the National Medical Ethics Committee of the Republic of Slovenia (0120-142/2021/2). Written informed consent was obtained from all patients/parents. This study was registered at ClinicalTrials.gov (NCT05123768).

## 3 Results

### 3.1 Clinical characteristic of our cohort

Our final cohort consisted of 136 children with a median age of 15 years (IQR 12–17 years, range 4–20 years), and 68 patients were male (50.0%). The basic clinical characteristics and comorbidity data of the patients are summarized in Table 1. We also present basic registry data of all patients entered into the SRCP in Table 1 for easier assessment of selection bias.

**Table 1:** Clinical characteristics of the cohort compared to the whole registry.

Characteristics	Cohort	Whole Registry
No. of patients	N = 136	N = 685
Age, years, median (IQR), [range]	15 (12–17), [4-20]	
Sex, No. (%)		
Female	68 (50.00)	305 (44.60)
Male	68 (50.00)	380 (55.40)
CP type, No. (%)		
Spastic	116 (85.29)	611 (89.33)
Unilateral	34 (25.00)	204 (29.82)
Bilateral	82 (60.29)	404 (59.06)
Unknown	/	3 (0.44)
Dyskinetic	17 (12.50)	63 (9.21)
Dystonic	13 (9.56)	31 (4.53)
Choreo-athetotic	1 (0.74)	6 (0.87)
Unknown	3 (2.21)	12 (1.75)
Ataxic	3 (2.21)	10 (1.32)
GMFCS, No.%		
Level I	49 (36.03)	266 (38.89)
Level II	26 (19.12)	119 (17.25)
Level III	11 (8.09)	50 (7.31)
Level IV	30 (22.06)	121 (17.69)
Level V	20 (14.71)	112 (16.37)
Unkown	/	16 (2.34)
Comorbidities, No. (%)		
Epilepsy	55 (40.44)	
Cognitive impairment	75 (55.15)	
Visual impairment	64 (47.06)	
Hearing impairment	9 (6.62)	
Autism spectrum disorder	8 (5.88)	
Speech and language disorders	53 (38.97)	
Behaviour and emotional disorders	14 (10.29)	
Attention deficit disorders	15 (11.03)	
Feeding difficulties	15 (11.03)	
Perinatal history		
Birth, No.(%)	88 (64.71)	549 (80.26)
Vaginal birth	18 (13.24)	103 (15.06)
Elective cesarean section	28 (20.59)	4 (0.58)
Urgent cesarean section	2 (1.47)	30 (4.39)
Unknown		292 (42.69)
Birth types, No. (%)	132	
Number of pregnancies	114 (83.82)	141 (20.61)
Singletons	21 (15.44)	167 (24.42)
Twins	8 (5.88)	61 (8.92)
Both have CP	13 (9.56)	22 (3.22)
Only one has CP	1 (0.74)	
Triplets	/	
Unknown		
Neonatal history		
Gestational age, weeks, median (IQR),[range]	37(31–39), [22-41]	
Gestational age category, No.(%)		
> = 37	69 (50.74)	
32–36	31 (22.79)	
27–31	25 (18.38)	
<27	10 (7.35)	
Unknown	1 (0.74)	
Birth weight, g, median (IQR), [range]	2,565 (1,630-3,292.5), [540-4,850]	
Birth weight category, No. (%)		
<1,000	12 (8.82)	
1,000–1,499	17 (12.50)	
1,500–1,999	15 (11.03)	
2,000–2,499	18 (13.24)	
≥2,500	66 (48.53)	
Unknown	8 (5.88)	
Apgar score at 5 min, No.(%)		
> = 7	96 (70.59)	
<7	24 (17.65)	
Unknown	16 (11.76)	

Of the 136 children, 116 (85%) had spastic CP, 17 (13%) had dyskinetic CP, and three (2%) had ataxic CP. All children were classified into five categories according to their current gross motor function using the Gross Motor Function Classification System (GMFCS) tool. Among them, 49 (36%) were Level I, 26 (19%) were Level II, 11 (8%) were Level III, 30 (22%) were Level IV, and 20 (15%) were Level V. The prevailing comorbidities were cognitive impairment in 75 (55%) patients, visual disturbances in 64 (47%) patients, epilepsy in 55 (40%) patients, and speech and language disorders in 53 (39%) patients.

According to perinatal history, 21 (15%) children were born after twin pregnancy, and one (1%) child was born after a triplet pregnancy. Among the included twins, there were four twin pairs in which both children had CP. The median gestational age at birth was 37 weeks (range, 22–41 weeks); 66 (49%) children were delivered preterm, and 28 (21%) children were born after an urgent cesarean section. The median birth weight was 2,565 g (range, 540–4,850 g). Apgar score data were available for 120 (88%) children; 24 (18%) children had Apgar scores lower than 7 at 5 min.

**Figure 1:**
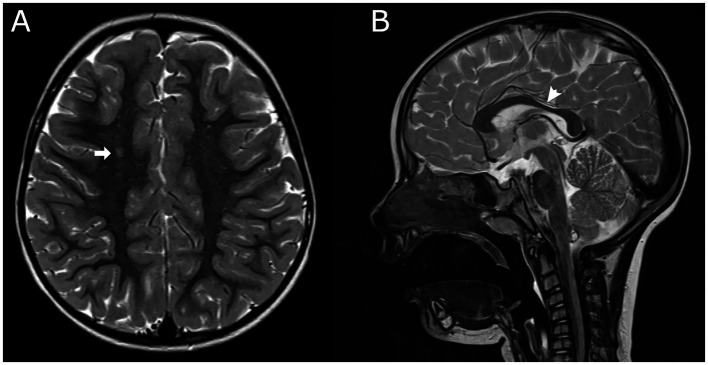
Brain MRI of Patient no. 1. **(A)** Axial T2-weighted image shows a small focus of increased signal in the right frontal centrum semiovale (arrow). Additional subtle white matter hyperintensities were present in the left peritrigonal region and right parietal subcortical white matter (not shown). **(B)** Sagittal T2-weighted image demonstrates mild thinning of the dorsal third of the corpus callosum (arrowhead). No additional pathological intracranial or spinal abnormalities were identified. The neuroradiological interpretation was consistent with mild chronic changes of presumed ischemic etiology. For data on perinatal history, see Table 4. Genetic testing revealed a *de novo* pathogenic variant in the ATL1 gene, consistent with a diagnosis of spastic paraplegia type 3A.

We gathered the neuroimaging data for 123 children (90%) from the SRCP. The latest brain MRI was utilized for classification where available, and neonatal ultrasound (US) was employed in patients without a brain MRI. For the purposes of this study, we did not re-review the original imaging; instead, neuroimaging findings were extracted from the SRCP. Most children (108, 79%) had some abnormalities, and only 8 (6%) children had normal imaging results. White matter lesions were the most commonly reported abnormalities, occurring in 79 patients (58%). An example of predominately white matter lesions in a patient with confirmed pathologic variant in ATL1 gene (patient no. 1) is shown in Figure 1. The neuroimaging data are summarized in Table 2.

**Table 2:** Neuroimaging data^*^.

Neuroimaging findings	*N* (%)
Predominantly white matter lesion	79 (58.1)
Periventricular leukomalacia	37 (27.2)
Sequelae of IVH or periventricular haemorrhagic infarction	9 (6.6)
Combination of PVL and IVH sequelae	11 (8,1)
Not Subclassified	22 (16.2)
Predominantly gray matter lesion	10 (7.4)
Basal ganglia/thalamus lesions	6 (4.4)
Cortico-subcortical lesions only not covered under C3	0 (0.0)
Arterial infarctions	3 (2.2)
Not Subclassified	1 (0.7)
Maldevelopments	10 (7.4)
Disorders of cortical formation	5 (3.7)
Other maldevelopments	3 (2.2)
Not Subclassified	2 (1.5)
Miscellaneous	9 (6.6)
Normal	8 (5.9)
Unknown	7 (5.2)

*Neuroimaging data were primarily classified using MRI findings, where available. In cases where MRI was not performed, US findings were used, provided that US had been conducted. For classification, MRI classification system—MRICS was used (41).

### 3.2 Genetic findings

Whole-exome sequencing (WES) was performed for all 136 patients. For this analysis, a gene panel of 110 CP-associated genes was analyzed. After evaluation by a clinical geneticist and laboratory scientist, the discovered variants were classified into one of four groups: pathogenic variant (P), likely pathogenic variant (LP), variant of unknown significance (VUS), and negative result (N), using ACMG recommendations for reporting of Incidental Findings in Clinical Exome and Genome Sequencing (21). To determine the origin of the variant, LP and VUS cases required further evaluation using segregation studies in both parents. The results showed that in 6/13 patients, the variant was *de novo*, in 5/13 it was inherited from one parent, and in 1/13 from both parents. The final genetic diagnostic yield is shown in Table 3.

**Table 3:** Final genetic diagnostic outcome based on WES methods.

Finding	N (%)
Positive genetic finding	9 (6.62)
Pathogenic	5 (3.68)
Likely pathogenic	1 (0.74)
VUS, likely clinically significant	3 (2.21)
VUS, likely clinically insignificant	4 (2.94)
Negative finding	123 (90.44)

We identified pathogenic variants, likely pathogenic variants, or ‘de novo’ VUS (VUS-P; variant of unknown significance—likely clinically significant) in nine patients, with the following genes being affected: *ATL1*, *CTNNB1*, *DYRK1*, *KMT2A*, *PROC*, *SPAST*, *ZC4H2*, and *ZSWIM6*. All nine patients showed clinical features consistent with their genetic diagnosis, which was further confirmed by clinical evaluations conducted by a geneticist and a neurologist. The VUS-P category was used to avoid reporting insignificant findings of rare variants that, according to ACMG criteria, are reported solely due to their rarity or absence of frequency data in population databases. VUS-P indicated that the mutation mechanism, gene, and the child’s clinical signs and symptoms matched; however, because these were most often missense mutations and had never been previously reported in the literature, we were unable to increase the confidence level of the finding to a likely pathogenic variant.

The diagnostic yield of clinically significant genetic variants was nine of 136 cases (6.6%). All pathogenic, likely pathogenic, and VUS-P variants were included in the calculation of the diagnostic yield. In contrast, VUS without clinical correlation to the child’s phenotype were excluded and not considered in the diagnostic yield calculation.

The clinical characteristics and genetic data of the patients are shown in Table 4. Most patients had spastic CP (7/9), mostly bilateral (4/7). Six patients were born full-term. Most (8/9) were exposed to prenatal and perinatal risk factors. Two patients in our cohort were twins (patients no. 2 and 3), and one patient was born as a part of a set of twins where the twin sibling was not affected by CP (patient no. 9). Neuroimaging studies identified white matter lesions in 7/9 patients, while two patients had a normal MRI. Among the associated comorbidities, only one patient had epilepsy, while the majority (6/9) had intellectual disabilities. “Red flags” were present in 6/9 patients.

**Table 4:** Clinical characteristics of patients with clinically significant genetic findings.

No.	Gene; diagnosis ACMG category; ACMG criteria Zygosity tipe Variant origin	Gender	CP subtype; GMFCS	Family and perinatal history	Weeks of gestation; mode of delivery	Radiologic findings	Associated disease	Red flags^1^
1	*ATL1* (NM_001127713.1):c.715C > T; Spastic paraplegia 3A P; PS4, PM5, PM2, PP3 Het *De novo*	F	Spastic bilateral; I	FH: Neg. PH: Maternal Rh isosensibilization and gestational diabetes, abnormal neurological signs and hyperbilirubinemia after birth	38; vaginal birth	MRI: Predominantly white matter changes, bilaterally, thinner corpus callosum, see Figure 1	Congenital megaureter	/
2	*CTNNB1* NM_001904.4:c[688dup];[=]; Neurodevelopmental disorder with spastic diplegia and visual defects P; PVS1, PM2, PP1 het De novo	M	Dystonic diskinetic; III	FH: Mother has Bainbridge-Ropers syndrome. Two half-sisters have Bainbridge-Ropers syndrome and SCN1A-related epilepsy PH: Twin pregnancy, premature birth, reanimation at birth, HIE, NICU admission	31; vaginal	US: Predominantly white matter changes, bilaterally MRI not performed	Moderate intelectual disability	FH: twin with CP
3	*CTNNB1* NM_001904.4:c[688dup];[=]; Neurodevelopmental disorder with spastic diplegia and visual defects P; PVS1, PM2, PP1 het De novo	M	Dystonic diskinetic; III	FH: Same as patient no. 2 PH: Twin pregnancy, premature birth, HIE, NICU admission, hyaline membrane disease	31; vaginal	US: Predominantly white matter changes, bilaterally MRI not performed	Moderate intelectual disability	FH: twin with CP
4	*KMT2A* (NM_001197104.2):c.3960del(p Thr1322ProfsTer34); Wiedemann-Steiner syndrome P; PVS1, PM2 Het De novo	F	Spastic bilateral; I	FH: Neg. PH: Uneventful pregnancy and birth history, NICU admission, dysmorphic features	39; vaginal	MRI: Predominantly white matter changes -bilateral PVL, corpus callosum agenesis	Intelectual disability, speech and feeding disorder, congenital hip dysplasia	Dysmorphic features
5	*SPAST* (NM_014946.4):c.1276C > T (p Leu426Phe); Spastic paraplegia type 4 P; PS4_Mod, PM2, PM5, PP2, PP3 Het Unknown	M	Spastic bilateral; I	FH: Neg. PH: Weak fetal movements, unprogressive labor, swallowing difficulty and hyperbilirubinemia after birth	40; urgent CS	MRI: Predominately white matter changes—bilateral PVL, normal spinal MRI	Speech fluency disorder, specific learning difficulties	Paraparesis
6	*DYRK1* (NM_001396.4):c.1316_1317delCTinsTCATAC; AD intellectual disability type 7 LP; PVS1, PM2 Het De novo	F	Spastic bilateral; I	FH: Neg. PH: IUGR, hyperbilirubinemia, NICU admission—perinatal SGB sepsis and pneumonia	38; vaginal	MRI: Normal	Epilepsy, moderate intellectual disability, speech disorder, hiatal hernia, microcephaly	Normal MRI
7	*PROC* (NM_000312.4):c.119G > A(;)1181G > A; Protein C deficiency VUS-P; 1. PM2, 2. PM2 c-het Parents carriers	M	Spastic unilateral; I	FH: Sudden cardiac arrest in many family members PH: bilateral ventriculomegaly at 34 GW, abnormal neurological signs after birth, obstructive hydrocephalus in the first month of life	38; vaginal	MRI: Predominantly white matter changes—Combination of cystic PVL and IVH sequelae (left), ventricular drainage	Attention disorder	/
8	*ZC4H2* (NM_018684.4):c.170A > C; Wieacker Wolff syndrome VUS-P; PM2, PP2, PP3 Hemi Unknown	M	Spastic unilateral; II	FH: Family member with CP PH: uneventful	40; vaginal	MRI: Normal	Intelectual disability, speech and language disorder	Uneventful PH, family member with CP, normal MRI
9	*ZSWIM6* (NM_020928.2):c.1022A > G(p. Asn341Ser); Neurodevelopmental disorder with movment abnormalities, abnormal gaint and autistic features VUS-P; PP3 Het De novo	F	Spastic unilateral; I	FH: Neg. PH: twin pregnancy, IUGR, NICU admission-meconium aspiration, IVH, hydrocephalus, outer and VP shunt	35; elective CS	MRI: Predominantly white matter changes, bilateral cystic PVL	Intelectual disability, strabismus, learning disorder, short stature	/

P, pathogenic variant; LP, likely pathogeniv variant; VUS-P variant of unknown significance—likely clinically significant; het, heterozygous; hom, homozygous; c-het, compound heterozygous; hemi, hemizygous; F, female; M, male; FH, family histrory; PH, perinatal histroy; Neg., negative; CS, cesarean section; IUGR, intrauterine growth restriction; LP, likely pathogenic; Neg., negative; NICU, neonatal intensive care unit.

1 Red flags are defined in the methods section.

## 4 Discussion

Cerebral palsy is the most common physical disability of early childhood and is traditionally associated with perinatal complications (1). Recent advancements and improved accesibility of genetic diagnostics have significantly enhanced our understanding of the genetic factors influencing the development of CP (6, 22, 23).

This study aimed to investigate the genetic etiology of CP in Slovene children. Slovenia is a Central European country with a relatively ethnically homogeneous population of approximately 2.1 million (24) and averages 18,300 births annualy over the past 5 years (24). Slovenia has a universal publicly funded healthcare system, in which every child is assigned a primary pediatrician who refers them to specialist care when needed. Children with suspected CP are typically referred to child neurologists, who are available at two tertiary centers and secondary hospitals across Slovenia. Genetic testing and counseling are predominantly conducted at the University Medical Centre Ljubljana. The SRCP is managed from the University Children’s Hospital in Ljubljana, where data on all children with CP in Slovenia are centrally collected.

In our study, we searched for the genetic etiology of CP in children in whom physicians did not suspect a genetic cause, or in whom limited genetic testing with negative results was previously performed. This is a different approach from other studies that have concentrated on specific subgroups of patients with CP (25, 26) or on all CP patients with available blood samples (8, 12, 13). In our cohort, the yield of genetic testing was 6.6%, revealing six children with confirmed genetic diagnoses and 3 children with probable genetic diagnoses that underlie CP, which emphasizes the importance of dedicated NGS panels in evaluation of patients with CP. Our relatively low diagnostic yield, compared to other studies (9, 10), can be attributed to the fact that many children from SRCP already had confirmed genetic diagnoses and were thus excluded from our study. Nevertheless, our findings highlight a prevalent clinical decision-making dilemma: determining whether to refer children with unsuspicious history for genetic etiology for genetic testing and whether to retest those who have previously recieved negative results.

Estimating the true prevalence of genetic etiology in CP is challenging because of inconsistent approaches to CP diagnosis among published studies and variations in study design (22). Consequently, it is difficult to compare our findings to other studies. Pham et al. (22) conducted a systematic review on phenotypic definition and case ascertainment in genetic studies of CP, including 57 studies. They found limited compliance with the SCPE international guidelines on defining CP, hindering result comparison among genetic studies of CP and potentially leading to misclassification of unrelated neurological conditions as CP. They noticed that many studies included children under 4 years of age, which further increased the potential for misclassification. They also noted that some studies have focused on specific subgroups of patients, such as idiopathic CP (22, 27). In our study, we included children aged ≥ 4 years, and CP diagnosis was re-evaluated before inclusion.

Some “red flags,” which are clinical characteristics pointing to genetic or metabolic etiology of CP have recently been defined (16, 18, 25). Although the presence of “red flags” aids in suspecting an underlying genetic diagnosis in a clinical setting, it is essential to note that certain red flags may exclude a diagnosis of CP. For instance, progressive symptoms or isolated hypotonia contradict the updated 2014 SCPE decision tree (17). Furthermore, the absence of red flags does not exclude a genetic etiology. In our study, out of nine children with definite or probable genetic etiology, we identified three children with absence of red flags, while six presented with the red flag symptoms.

Neuroimaging findings play a crucial role in identifying the genetic basis of CP (28). Children with normal MRI findings or brain malformations often have an underlying genetic cause (28). In our cohort, neuroimaging data were not available for all the patients; however, of the nine patients with positive genetic findings, two had normal MRI findings, while seven were classified as having predominantly white matter changes, which were probably linked to supposed perinatal adverse events and therefore limited the clinicians’ push for further diagnostic testing. We presented the brain MRI of a patient (Patient no. 1, Figure 1) carrying a pathogenic variant in the ATL1 gene, consistent with spastic paraplegia type 3A. The scan showed predominately white matter lesions and corpus callosum thinning, interpreted as mild chronic changes of presumed ischaemic aetiology. Although a thin corpus callosum has been reported in some patients with this condition (29), white-matter abnormalities have not previously been described or associated with spastic paraplegia type 3A (30).

Recent studies have shown that a subset of neonates with clinical signs of hypoxic–ischemic encephalopathy may, in fact, have an underlying genetic disorder that mimics or predisposes to encephalopathy (31–33). This underscores the potential value of early genetic testing in the neonatal period, particularly when perinatal history is ambiguous or sentinel events are present. Compared to retrospective assessment in older children with CP—where documentation is often incomplete and medical histories span several years—genetic evaluation in neonates allows for interpretation within a clearer and more complete clinical context.

CP is sometimes the initial diagnosis given to patients who later develop progressive neurological disorders. For example, patient no. 5 in Table 4 was initially diagnosed with bilateral spastic CP GMFCS I, as mild spasticity, jerky reflexes, and clonus were observed in his lower limbs. However, our study revealed a pathogenic variant in *SPAST* gene, leading to a revised diagnosis of spastic paraplegia type 4, which is a progressive disease (34). According to the International Cerebral Palsy Genomics Consortium (35), CP is primarily diagnosed based on clinical manifestations rather than etiology. Consequently, in patients in which the clinical presentation remains stable, the original diagnosis of CP should not be changed based on findings from brain imaging, metabolic screening, or genetic etiology (35). Our patient met the criteria for CP diagnosis at evaluation, although over time, the progression of the disease will probably become obvious, which will exclude the diagnosis of CP and he will be excluded from SRCP. Such cases highlight the importance of early genetic testing to ensure accurate diagnosis and appropriate follow-up.

Identifying the genetic cause of motor problems in patients diagnosed with CP is also important because of the possibility of targeted therapies for genetic and metabolic diseases, as some are already available or are being developed (36). Our study identified two twins with CTNNB1 syndrome (37), for which considerable effort has been made to develop gene replacement therapy by Miroševič and colleagues at the CTNNB1 foundation (38, 39).

Interestingly, our study found that most patients with clinically significant genetic variants had milder forms of CP—6/9 patients were GMFCS I, which may indicate underdiagnosis in this subgroup. Notably, our cohort contained a higher number of patients with GMFCS I (36%), which could partially contribute to this pattern.

Our study consisted of a patient population in which 49% of the children were born prematurely. Of the nine patients with positive genetic findings, 6/9 were born full-term, while three were delivered preterm all due to twin pregnancy. A study conducted by Takezawa et al. focused on a specific subgroup of CP patients who were born full-term and exhibited normal or nonspecific findings on brain MRI (26). The researchers identified pathogenic or likely pathogenic candidate variants in nine out of 17 cases (53%). They concluded that a higher percentage of full-term children with CP can be attributed to genetic factors compared to the general population of patients with CP (26), which is consistent with our findings.

Finally, the accessibility of genetic diagnostics has grown considerably, leading to the identification of an expanding number of cases in which CP can be definitively or probably attributed to genetic factors. Therefore, we suggest that the presence of red flags should always prompt comprehensive genetic testing. It is crucial to note that children with a genetic etiology of CP might not exhibit these red flags—therefore, when faced with a case of etiologically unexplained CP, genetic testing should be considered. In our study, we offered an inclusive and comprehensive approach to genetic testing in patients with CP, resulting in the identification of nine patients with definite or probable genetic diagnoses, of which three exhibited no red flags. Although one could argue that our yield of genetic testing was low (6.6%) compared to other studies (9, 10), all genetic findings and genetic counselling were very important to all families participating in the study.

### 4.1 Strengths and limitations

The strength of our study is the relatively large cohort of patients compared to other similar studies. We used an inclusive approach when enrolling patients, as we offered genetic evaluation to all Slovenian children with CP without an established genetic diagnosis, without other exclusion criteria. The diagnosis of CP was reevaluated at enrollment. All the genetic findings were carefully interpreted by a clinical geneticist.

An important limitation of our study was the inability to accurately estimate the prevalence of genetic etiology in the entire CP population, as the study relied on voluntary participation. There was a potential bias in patient selection, as families of children with an unknown etiology may have been more keen on participating, as well as families of children with milder forms of CP, as these children are more likely to have their own families in the future. We believe that further larger prospective studies including the whole population of children with CP would provide a better approximation of the true prevalence of genetic etiology in CP.

Our findings are limited by the gene panel used, which was based on CP-associated genes listed on the HPO website in 2021. We acknowledge that many additional genes have since been linked to CP phenotypes. Furthermore, CNV analysis was not performed. These aspects may have contributed to underestimating the diagnostic yield. We recognize that broader panels and reanalysis in the future may improve diagnostic rates.

Another limitation of our study is that information on prior negative genetic testing was not systematically available for all included patients. Although we made efforts to gather this information, it was not previously collected in the SRCP, which limited our ability to assess how many participants had undergone prior testing without a diagnosis or whether reinterpretation could have yielded new findings. Recognizing the importance of this information, we plan to systematically include and prospectively collect prior genetic testing data in the SRCP in the future.

Another limitation of our study was that it included only patients from Slovenia. Because Slovenia is a small country, our number of participants is limited, and as a result, we found only a small number of patients with positive genetic finding, and those findings were heterogenous.

### 4.2 Future perspectives

Research like ours significantly contributes to the understanding of genetic factors in a complex condition like CP. Understanding the genetic etiology promises a broader range of therapeutic possibilities for children with CP, with targeted therapies including gene therapy, as well as new therapeutic options developed through drug repurposing (40). Our study reconfirms the previously suggested claim that a significant number of patients with CP actually carry an underlying genetic disease that could potentially benefit from such treatment. These findings suggest the importance of genetic screening for all patients with CP, not only for those with suggestive clinical signs, using a dedicated NGS CP panel.

## 5 Conclusion

In conclusion, definite or probable genetic etiology in children diagnosed with CP can be readily identified with appropriate genetic testing and is present in a significant number of patients with CP. Uncovering the underlying genetic mechanisms of the disease offers potential for targeted personalized treatment options. The presence of red flags in children with CP should always prompt comprehensive genetic testing, while we advocate offering genetic testing to all children with etiologically unexplained CP, even in the absence of red flags. Further prospective studies are warranted to support our recommendations and better understand the genes that are most commonly affected in patients with CP.

## Data Availability

The raw data supporting the conclusions of this article will be made available by the authors, without undue reservation.

## Footnotes

^1^ hpo.jax.org

## References

[1] Graham HK Rosenbaum P Paneth N Dan B Lin JP Damiano DL. Cerebral palsy. Nat Rev Dis Primers. (2016) 2:15082. doi: 10.1038/nrdp.2015.82, PMID: 27188686 PMC9619297

[2] Cans C. Surveillance of cerebral palsy in Europe (SCPE) reply. Dev Med Child Neurol. (2001) 43:575. doi: 10.1017/S001216220123104111508929

[3] Sellier E Platt MJ Andersen GL Krägeloh-Mann I de la Cruz J Cans C. Decreasing prevalence in cerebral palsy: a multi-site European population-based study, 1980 to 2003. Dev Med Child Neurol. (2016) 58:85–92. doi: 10.1111/dmcn.12865, PMID: 26330098

[4] McIntyre S Taitz D Keogh J Goldsmith S Badawi N Blair E. A systematic review of risk factors for cerebral palsy in children born at term in developed countries. Dev Med Child Neurol. (2013) 55:499–508. doi: 10.1111/dmcn.12017, PMID: 23181910

[5] Ellenberg JH Nelson KB. The association of cerebral palsy with birth asphyxia: a definitional quagmire. Dev Med Child Neurol. (2013) 55:210–6. doi: 10.1111/dmcn.12016, PMID: 23121164

[6] Fahey MC Maclennan AH Kretzschmar D Gecz J Kruer MC. The genetic basis of cerebral palsy. Dev Med Child Neurol. (2017) 59:462–9. doi: 10.1111/dmcn.13363, PMID: 28042670

[7] Friedman JM van Essen P Karnebeek CDM. Cerebral palsy and related neuromotor disorders: overview of genetic and genomic studies. Mol Genet Metab. (2021) 137:399–419. doi: 10.1016/j.ymgme.2021.11.001, PMID: 34872807

[8] Moreno-De-Luca A Millan F Pesacreta DR Elloumi HZ Oetjens MT Teigen C. Molecular diagnostic yield of exome sequencing in patients with cerebral palsy. Obstet Gynecol Surv. (2021) 76:399–401. doi: 10.1097/01.ogx.0000767212.46634.bb PMC7856544 33528536

[9] Gonzalez-Mantilla PJ Hu Y Myers SM Finucane BM Ledbetter DH Martin CL. Diagnostic yield of exome sequencing in cerebral palsy and implications for genetic testing guidelines: a systematic review and Meta-analysis. JAMA Pediatr. (2023) 177:472–8. doi: 10.1001/jamapediatrics.2023.0008, PMID: 36877506 PMC9989956

[10] Srivastava S Lewis SA Cohen JS Zhang B Aravamuthan BR Chopra M. Molecular diagnostic yield of exome sequencing and chromosomal microarray in cerebral palsy: a systematic review and Meta-analysis. JAMA Neurol. (2022) 79:1287–95. doi: 10.1001/jamaneurol.2022.3549, PMID: 36279113 PMC9593320

[11] van Eyk CL Fahey MC Gecz J. Redefining cerebral palsies as a diverse group of neurodevelopmental disorders with genetic aetiology. Nat Rev Neurol. (2023) 19:542–55. doi: 10.1038/s41582-023-00847-6, PMID: 37537278

[12] McMichael G Bainbridge MN Haan E Corbett M Gardner A Thompson S. Whole-exome sequencing points to considerable genetic heterogeneity of cerebral palsy. Mol Psychiatry. (2015) 20:176–82. doi: 10.1038/mp.2014.189, PMID: 25666757

[13] Fehlings DL Zarrei M Engchuan W Sondheimer N Thiruvahindrapuram B MacDonald JR. Comprehensive whole-genome sequence analyses provide insights into the genomic architecture of cerebral palsy. Nat Genet. (2024) 56:585–594. doi: 10.1038/s41588-024-01686-x 38553553

[14] Pearson TS Gupta N San Sebastian W Imamura-Ching J Viehoever A Grijalvo-Perez A. Gene therapy for aromatic L-amino acid decarboxylase deficiency by MR-guided direct delivery of AAV2-AADC to midbrain dopaminergic neurons. Nat Commun. (2021) 12:4251. doi: 10.1038/s41467-021-24524-8, PMID: 34253733 PMC8275582

[15] Tai CH Lee NC Chien YH Byrne BJ Muramatsu SI Tseng SH. Long-term efficacy and safety of eladocagene exuparvovec in patients with AADC deficiency. Mol Ther. (2022) 30:509–18. doi: 10.1016/j.ymthe.2021.11.005, PMID: 34763085 PMC8822132

[16] Ashwal S Russman BS Blasco PA Miller G Sandler A Shevell M. Practice parameter: diagnostic assessment of the child with cerebral palsy: report of the quality standards Subcommittee of the American Academy of neurology and the practice Committee of the Child Neurology Society. Neurology. (2004) 62:851–63. doi: 10.1212/01.WNL.0000117981.35364.1B, PMID: 15037681

[17] Surveillance of Cerebral Palsy in Europe. Surveillance of cerebral palsy in Europe: a collaboration of cerebral palsy surveys and registers. Dev Med Child Neurol. (2000) 42:816–24. doi: 10.1017/s0012162200001511 11132255

[18] Lee RW Poretti A Cohen JS Levey E Gwynn H Johnston MV. A diagnostic approach for cerebral palsy in the genomic era. NeuroMolecular Med. (2014) 16:821–44. doi: 10.1007/s12017-014-8331-9, PMID: 25280894 PMC4229412

[19] Hakami WS Hundallah KJ Tabarki BM. Metabolic and genetic disorders mimicking cerebral palsy. Neurosciences. (2019) 24:155–63. doi: 10.17712/nsj.2019.3.20190045, PMID: 31380813 PMC8015517

[20] Poplin R Ruano-Rubio Valentin DePristo MA Fennell TJ Carneiro MO Van der Auwera GA. (2018). Scaling accurate genetic variant discovery to tens of thousands of samples. BioRxIV. 1–22.

[21] Richards S Aziz N Bale S Bick D das S Gastier-Foster J. Standards and guidelines for the interpretation of sequence variants: a joint consensus recommendation of the American College of Medical Genetics and Genomics and the Association for Molecular Pathology. Genet Med. (2015) 17:405–24. doi: 10.1038/gim.2015.30, PMID: 25741868 PMC4544753

[22] Pham R Mol BW Gecz J MacLennan AH MacLennan SC Corbett MA. Definition and diagnosis of cerebral palsy in genetic studies: a systematic review. Dev Med Child Neurol. (2020) 62:1024–30. doi: 10.1111/dmcn.14585, PMID: 32542675

[23] Jin SC Lewis SA Bakhtiari S Zeng X Sierant MC Shetty S. Mutations disrupting neuritogenesis genes confer risk for cerebral palsy. Nat Genet. (2020) 52:1046–56. doi: 10.1038/s41588-020-0695-1, PMID: 32989326 PMC9148538

[24] Statistical Office Republic of Slovenia (2025). Available online at: https://pxweb.stat.si/SiStat/en (Accessed July 4, 2025).

[25] Zouvelou V Yubero D Apostolakopoulou L Kokkinou E Bilanakis M Dalivigka Z. The genetic etiology in cerebral palsy mimics: the results from a Greek tertiary care center. Eur J Paediatr Neurol. (2019) 23:427–37. doi: 10.1016/j.ejpn.2019.02.001, PMID: 30799092

[26] Takezawa Y Kikuchi A Haginoya K Niihori T Numata-Uematsu Y Inui T. Genomic analysis identifies masqueraders of full-term cerebral palsy. Ann Clin Transl Neurol. (2018) 5:538–51. doi: 10.1002/acn3.551, PMID: 29761117 PMC5945967

[27] Segel R Ben-Pazi H Zeligson S Fatal-Valevski A Aran A Gross-Tsur V. Copy number variations in cryptogenic cerebral palsy. Neurology. (2015) 84:1660–8. doi: 10.1212/WNL.0000000000001494, PMID: 25817843

[28] Mochida GH Walsh CA. Genetic basis of developmental malformations of the cerebral cortex. Arch Neurol. (2004) 61:637–40. doi: 10.1001/archneur.61.5.637, PMID: 15148137

[29] OMIM (2025). Spastic paraplegia 3A, autosomal dominant. Available online at: https://omim.org/entry/182600#diagnosis (Accessed July 4, 2025).

[30] Stevanin G. (2021). Spastic Paraplegia 11 Summary Genetic counseling Suggestive Findings. GeneReviews. Published online 2021:1-15. Available online at: https://www.ncbi.nlm.nih.gov/books/NBK1827/pdf/Bookshelf_NBK1827.pdf (Accessed July 4, 2025).

[31] Lee S Kim SH Kim HD Lee JS Ko A Kang HC. Genetic diagnosis in neonatal encephalopathy with hypoxic brain damage using targeted gene panel sequencing. J Clin Neurol. (2024) 20:519–28. doi: 10.3988/jcn.2023.0500, PMID: 39227335 PMC11372210

[32] Parobek CM Zemet R Shanahan MA Burnett BA Mizerik E Rosenfeld JA. Clinical exome sequencing uncovers genetic disorders in neonates with suspected hypoxic–ischemic encephalopathy: a retrospective analysis. Clin Genet. (2024) 106:95–101. doi: 10.1111/cge.14522, PMID: 38545656 PMC11147704

[33] Woodward KE Murthy P Mineyko A Mohammad K Esser MJ. Identifying genetic susceptibility in neonates with hypoxic-ischemic encephalopathy: a retrospective case series. J Child Neurol. (2023) 38:16–24. doi: 10.1177/08830738221147805, PMID: 36628482

[34] Spastic Paraplegia 4 (2017). GeneReviews® - NCBI Bookshelf. Available online at: https://www.ncbi.nlm.nih.gov/books/NBK1160/ [Accessed August 18, 2023].

[35] MacLennan AH Lewis S Moreno-De-Luca A Fahey M Leventer RJ McIntyre S. Genetic or other causation should not change the clinical diagnosis of cerebral palsy. J Child Neurol. (2019) 34:472–6. doi: 10.1177/0883073819840449, PMID: 30963790 PMC6582263

[36] Kojima K Nakajima T Taga N Miyauchi A Kato M Matsumoto A. Gene therapy improves motor and mental function of aromatic l-amino acid decarboxylase deficiency. Brain. (2019) 142:322–33. doi: 10.1093/brain/awy331, PMID: 30689738 PMC6377184

[37] Miroševič Š Khandelwal S Sušjan P Žakelj N Gosar D Forstnerič V. Correlation between phenotype and genotype in CTNNB1 syndrome: a systematic review of the literature. Int J Mol Sci. (2022) 23:1–24. doi: 10.3390/ijms232012564, PMID: 36293418 PMC9604177

[38] CTNNB1 Syndrome (2019). CTNNB1 Foundation. Available online at: https://ctnnb1-foundation.org/gene-replacement-therapy-program/ (Accessed May 1, 2024).

[39] Osredkar D. (2022). ClinicalTrials.Gov: Genotype-Phenotype Correlations in Children and Adults With CTNNB1 Mutation ID NCT04812119. Available online at: https://clinicaltrials.gov/study/NCT04812119#study-overview (Accessed July 4, 2025)

[40] Shah S Dooms MM Amaral-Garcia S Igoillo-Esteve M. Current drug repurposing strategies for rare neurodegenerative disorders. Front Pharmacol. (2021) 12:768023. doi: 10.3389/fphar.2021.768023, PMID: 34992533 PMC8724568

[41] Himmelmann K Horber V De La Cruz J Horridge K Mejaski-Bosnjak V Hollody K. MRI classification system (MRICS) for children with cerebral palsy: development, reliability, and recommendations. Dev Med Child Neurol. (2017) 59:57–64. doi: 10.1111/dmcn.13166, PMID: 27325153

