# Supplementary material for: Unravelling genetic etiology of cerebral palsy: findings from a Slovenian pediatric cohort
Source: Front Neurol. 2025 Jul 23;16:1615449. doi: 10.3389/fneur.2025.1615449 (PMC12325036; doi:10.3389/fneur.2025.1615449)
Supplement: Supplementary file 1 [file Table_1.docx]

**Supplementary Material – Table 1**

**Gene panel**

| **Gene** | **NM #** | **Gene** | **NM #** | **Gene** | **NM #** | **Gene** | **NM #** | **Gene** | **NM #** | **Gene** | **NM #** | **Gene** | **NM #** |
| --- | --- | --- | --- | --- | --- | --- | --- | --- | --- | --- | --- | --- | --- |
| ACTB | NM_001101 | BCAP31 | NM_001256447 | ECHS1 | NM_004092 | ITPR1 | NM_001378452 | NTN1 | NM_004822 | SLC2A1 | NM_006516 | WIPI2 | NM_015610 |
| ADAR | NM_001111 | BCL11A | NM_022893 | ERCC8 | NM_000082 | KANK1 | NM_015158 | OTUD6B | NM_016023 | SLC5A6 | NM_021095 | ZC4H2 | NM_018684 |
| ADCY5 | NM_183357 | CACNA1A | NM_001127222 | EXOSC3 | NM_016042 | KCNC3 | NM_004977 | PAK3 | NM_002578 | SLC6A3 | NM_001044 | ZEB2 | NM_014795 |
| ADD3 | NM_016824 | CACNA1D | NM_001128840 | FBXO31 | NM_024735 | KCNQ2 | NM_172107 | PANK2 | NM_001386393 | SLCO2A1 | NM_005630 | ZSWIM6 | NM_020928 |
| ADNP | NM_001282531 | CAMTA1 | NM_015215 | FBXW11 | NM_001378974 | KDM5C | NM_004187 | PCBD1 | NM_000281 | SMARCB1 | NM_003073 |  |  |
| AGAP1 | NM_001037131 | CASK | NM_001367721 | FOXG1 | NM_005249 | KDM7A | NM_030647 | PCDH12 | NM_016580 | SPAST | NM_014946 |  |  |
| AHDC1 | NM_001371928 | CD99L2 | NM_031462 | GABRA2 | NM_000807 | KIDINS220 | NM_020738 | PCDH19 | NM_001184880 | SPG11 | NM_025137 |  |  |
| AIP | NM_003977 | CHD8 | NM_001170629 | GAD1 | NM_000817 | KIF1A | NM_001244008 | PCYT2 | NM_002861 | SPR | NM_003124 |  |  |
| ALDH3A2 | NM_000382 | COL4A1 | NM_001845 | GNAO1 | NM_020988 | KMT2A | NM_001197104 | PIGN | NM_176787 | SPTAN1 | NM_001130438 |  |  |
| ALK | NM_004304 | COL4A2 | NM_001846 | GNB1 | NM_002074 | KMT2B | NM_014727 | PNP | NM_000270 | STXBP1 | NM_001032221 |  |  |
| ALS2 | NM_020919 | CREBBP | NM_004380 | GP1BA | NM_000173 | L1CAM | NM_001278116 | PROC | NM_000312 | SYT1 | NM_005639 |  |  |
| AMPD2 | NM_001368809 | CTBP1 | NM_001012614 | GP1BB | NM_000407 | MAOB | NM_000898 | PURA | NM_005859 | TAF1 | NM_004606 |  |  |
| AP4B1 | NM_001253852 | CTNNB1 | NM_001904 | GPR101 | NM_054021 | MAST1 | NM_014975 | RAD51 | NM_002875 | TCF4 | NM_001083962 |  |  |
| AP4E1 | NM_007347 | CYP2U1 | NM_183075 | GRIN2B | NM_000834 | MECP2 | NM_001110792 | RBM10 | NM_005676 | TENM1 | NM_001163278 |  |  |
| AP4M1 | NM_004722 | DCC | NM_005215 | GSX2 | NM_133267 | MFN2 | NM_014874 | RFX2 | NM_000635 | TNR | NM_003285 |  |  |
| AP4S1 | NM_001128126 | DCX | NM_001195553 | HECW2 | NM_001348768 | MINPP1 | NM_004897 | RHOB | NM_004040 | TREX1 | NM_033629 |  |  |
| ARID2 | NM_152641 | DDC | NM_001082971 | HPCA | NM_002143 | NAA35 | NM_024635 | RNASEH2A | NM_006397 | TUBA1A | NM_006009 |  |  |
| ARX | NM_139058 | DDHD2 | NM_015214 | HPDL | NM_032756 | NALCN | NM_052867 |  |  |  |  |  |  |
